# Influence of Tooth Brushing and Previous Dental Visits on Dental Caries Status among Saudi Arabian Children

**DOI:** 10.3390/children10030471

**Published:** 2023-02-27

**Authors:** Sreekanth Kumar Mallineni, Abdullah Alassaf, Basim Almulhim, Sara Alghamdi

**Affiliations:** 1 Division for Globalization Initiative, Liaison Center for Innovative Dentistry, Graduate School of Dentistry, Tohoku University, Sendai 980-8575, Japan; 2 Center for Transdisciplinary Research (CFTR), Saveetha Dental College, Saveetha Institute of Medical and Technical Sciences, Saveetha University, Chennai 600077, India; 3 Pediatric Dentistry, Dr. Sulaiman Al Habib Hospital, Ar Rayyan, Riyadh 14212, Saudi Arabia; 4 Department of Preventive Dental Sciences, College of Dentistry, Majmaah University, Al Majmaah 11952, Saudi Arabia

**Keywords:** dental caries, primary dentition, Arabian children, tooth brushing, dental visit

## Abstract

Objective: To evaluate the dental caries status and its association with tooth brushing frequency and previous dental visits among Arabian children. Methods: Arabian school children attend a specialty pediatric dental clinic at Majmaah University, Saudi Arabia. Only children of Saudi origin with primary dentition were included in the study, and only one examiner was involved in the assessment and data collection. The parents of children completed a questionnaire to investigate possible explanatory variables for caries status, including tooth brushing frequency and previous dental visits. Dental caries were diagnosed according to the criteria recommended by the World Health Organization (2013). The comparison performed was based on sex, age, tooth brushing frequency, and previous dental visits. The descriptive statistics were carried out using SPSS (version 21.0) with a *p*-value of <0.05 significance. Results: The study involved 268 Saudi children with a mean age of 4.6 ± 1.1 years. The caries prevalence was 78.8%, and the mean dmft was 5.82 ± 4.48, while the overall mean scores for decayed, missing, and filled were 3.903 ± 3.69, 1.18 ± 1.69, and 0.73 ± 1.35, respectively. The mean dmft scores for the <3 years, 3.1–6 years, and >6 years old children were found to be 1.74, 6.58, and 4.58, respectively. Among the children, the females reported higher dmf scores (7.51 ± 4.18) compared to males (4.97 ± 4.39) with a statistically significant difference (*p* < 0.001). Fifty percent of the children had tooth brushing habits of once daily, followed by never (22.4%), twice daily (15.7%), and rarely (11.2%) brushed their teeth. Statistically significant correlations were found between the children’s dental caries status, age, and dental visits, while sex and tooth brushing did not find a correlation. Conclusion: The prevalence of dental caries among Arabian preschool children was higher. Mandibular second molars were commonly affected by dental caries, while mandibular central incisors were less frequently affected. The child’s age and frequency of dental checkups were positively related to the prevalence of dental caries.

## 1. Introduction

Dental caries is one of the most common chronic diseases in early childhood. It is considered a severe health problem globally [1]. Early childhood caries (ECC) is specified as the presence of one or more decayed (non-cavitated or cavitated lesions), missing (due to caries), or filled tooth surfaces in a child under the age of six. Any sign of smooth-surface caries in children under three years of age indicates severe early childhood caries (S-ECC) [2]. Awareness of the frequency and severity of ECC in children is crucial for its prevention. Control and reversion of ECC may be possible if the diagnosis is well-known at the initial stage of its occurrence, characterized by the presence of “white spots” on tooth enamel. When evaluating the severity of ECC between the ages of three and five, “dmf” ratings are used [1]. Numerous studies from the Kingdom of Saudi Arabia have reported a very high caries prevalence among preschool children [3,4]. Recent reports from Italy and Australia indicated unequal distributions in caries prevalence among preschoolers. Both studies reported a higher caries prevalence, and they postulated that the severity of caries in preschool children depends on ethnic and immigration groups [5,6].

In recent years, dental caries has become one of the most critical problems confronting Saudi Arabia’s public health [3]. There is a lacune in the research on early childhood colic (ECC) prevalence, the severity of symptoms, and treatment requirements in preschool-aged Saudi Arabian children [7,8]. Some of the researchers examined female participants in their studies, while others examined male participants; nevertheless, there were only a very small number of studies that examined both male and female participants together. The lack of data on caries patterns, the severity of carious lesions, and treatment needs in Saudi Arabia may be due to the difficulty in gaining access to patients in Saudi Arabian preschools [9]. Therefore, there is a continuing requirement to evaluate the severity and patterns of caries. A number of studies in Saudi Arabia have reported the correlation between dental caries and factors such as body mass index, dietary patterns, the level of education of parents, and oral hygiene [9,10,11,12,13]. However, there is still a need for data addressing the pattern and severity of caries in order to identify the extent and quality of caries preventive programs and treatment needs in preschool children in Saudi Arabia. The majority of research [9,10,11,12,13,14,15] concentrated on both mixed and permanent dentitions. However, no research has shown the influence of brushing frequency or the number of dental appointments one has had in the past on the presence or absence of dental caries. Henceforth, the present study aims to evaluate caries prevalence among Saudi children and to establish the association between dental caries status and tooth brushing frequency, and previous dental visits.

## 2. Methods

This cross-sectional study was performed from October 2020 to December 2021 and was described in agreement with the STROBE (Strengthening the Reporting of Observational Studies in Epidemiology) guidelines [16]. The Deanship of Scientific research at Majmaah University approved the study proposal and questionnaire on 1 October 2020. Informed consent was obtained from all the parents/guardians prior to the beginning of the study. The study population involved only Arabian-origin children attending the College of Dentistry, Majmaah University, AlZulfi, Saudi Arabia. The inclusion criteria of the study population were healthy children with primary dentition of Saudi origin. A single proportion formula was used to generate the sample size, and it was based on a confidence level of 95%, an expected prevalence of 80% [7], a precision of 0.05, and a design impact of 2. The desired sample size for the study was 252. Using prior research [9,10,11,12,13] and taking into account the cultural sensitivity of the study group, a structured, self-administered parental questionnaire was constructed. To ensure accuracy, the questionnaire was translated into Arabic and then back into English. Prior to the study, the face validity, practicality, and concept validity of the questionnaire were validated. Parents of the children who refused to consent and children with intellectual disabilities, systemic disease, and associated syndromes were excluded from the study. Dental caries diagnosed at the cavitation level mainly through visual inspection was used for diagnosis of dental caries, which was initially recommended by WHO (World Health Organization) [17]. One experienced examiner was involved in the study to avoid potential bias with excellent Kappa scores (0.9). The data was collected using a questionnaire regarding the study parameters, which required less than 5 min before the clinical session. The study was piloted and ensured that parents of the children should understand and respond to the questions feasibly. The responses from the pilot study were not included in the final analysis. The set of structured questionnaires was distributed to the parents or primary caregivers who accompanied the recruited children from the pediatric specialty dental clinic before the dental examinations. The questionnaires included name, gender (male or female), age (<3 years, 3.1–6 years, and >6 years), tooth brushing (once/twice/rarely/never), and previous dental visits (yes/no). The mean dmft scores were calculated and compared based on gender, age, tooth brushing frequency, and last dental visit. The tooth-based dmft mean scores were evaluated, and tooth type and arch-based tooth type comparisons were also made.

### Statistical Analysis

For categorical data, the number and percentage were reported. For numerical data, the mean (SD) was reported. Based on the normality of the data, the *t*-test was used to compare the two groups. The ANOVA test was used to reach more than two groups. Further linear regression was performed. The estimates with 95% CI were reported. All tests were two-sided at α = 0.05 level of significance. The multivariate linear regression analysis was used to establish an association between the four study variables’ mean dmft scores. For the regression analysis, male (gender), age (<3 years), twice (brushing frequency), and previous dental visits (yes) were used as reference points for the comparison of the data. All analyses were done using Statistical Package for Social Sciences (SPSS) software Version 21.0 (Armonk, NY, USA: IBM Corp).

## 3. Results

The study involved 268 children with primary dentition with a mean age of 4.6 ± 1.1 years. The majority were males (66.4%) and females (33.6%). Among the children involved in the study, 76.5% of the children belong to the 3.1–6 years age group, and below 3 years and above 6 years were 10.1% and 13.4%, respectively. Only 50.7% of the children in the study population brushed once daily, while 42% of children brushed twice daily, 11.2% rarely brushed their teeth, and 22.4% had never brushed their teeth before. Only 32.5% of children had visited the dentist before, and 67.5% visited the dentist for the first time during the study. The prevalence of carious lesions in the studied population was 78.8 percent, and the mean dmft for the population under research was 5.82 ± 4.48. While the mean scores for decayed, missing, and filled were 3.903 ± 3.69, 1.18 ± 1.69, and 0.73 ± 1.35, respectively. Among the study subjects, the females reported higher mean dmft scores (7.51 ± 4.18) compared to males (4.97 ± 4.39) with a statistically significant difference (*p* < 0.001). In the age group, the children of 3.1–6 years were observed with higher mean dmft scores of 6.58 ± 4.28 followed by above 6 years (4.58 ± 4.91) and below 3 years (1.74 ± 2.33), and the comparison was highly significant (*p* < 0.001). The children who brushed their teeth twice had lower dmft scores (3.09 ± 4.07). In contrast, children who never brushed before had higher dmft scores (7.5 ± 4.56) compared to those rarely touched (4.10 ± 4.38), and children who brushed once daily (6.06 ± 4.27) and the comparison of tooth brushing and dmft was statistically significant (*p* < 0.001). The children who visited the dentist before had high dmft scores (8.14 ± 3.12) compared to children who had never seen a dentist before (4.71 ± 4.61), and the comparison of a previous dental visit and dmft scores was statistically significant (*p* < 0.001). The comparison of the dmft based on sex, age, tooth brushing, and the previous dental visit are summarized in Table 1.

Based on tooth type, higher dmft scores were observed for tooth 85 (mandibular right second molar), while lower dmft scores were achieved for mandibular central incisors (teeth 71 and 81); see Table 2. The most commonly decayed tooth was the mandibular right second primary molar (85), and the less commonly decayed teeth were mandibular central incisors (71 and 81). The most commonly missing tooth due to dental caries was the maxillary right primary central incisor (tooth 51), and the less commonly missing tooth was the maxillary right primary canine (tooth 53). The teeth never missing due to caries were teeth 55, 63, 65, 73, 72, 71, 81, 82, and 83. The most frequently filled tooth was the mandibular right second primary molar, while less commonly filled teeth were primary maxillary canines (teeth 53 and 63). However, teeth 62, 72, 71, 81, and 82 were never filled in any of the children. The most commonly decayed tooth was the second primary molar in both maxillary and mandibular arches. The less widely decayed tooth was the left canine in the maxillary arch, while the primary central incisors were in the mandibular arch.

The gender comparison among the age groups should have mixed results. The dmft scores in the 3.1–6 years of age group showed statistical significance (*p* < 0.001), while gender comparison among <3 years and >6 years should be statistically non-significant (*p* > 0.05). The female subjects showed higher dmft scores than males in all age groups studied. The gender-based comparison among the various age groups is summarized in Table 3.

The comparison of mean dmft scores based on tooth type showed statistical significance (*p* < 0.05) for decayed, missing, and filled scores. Mandibular molars were commonly decayed teeth, followed by maxillary molars, maxillary incisors, maxillary canines, and mandibular incisors, with statistically significant differences (*p* = 0.004). Maxillary incisors were the most commonly missing teeth due to caries, while maxillary canines were less likely to be missing due to dental caries; however, mandibular canine and mandibular incisors were never missing due to dental caries in the study population. Mandibular molars were commonly filled teeth, followed by maxillary molars, maxillary incisors, and maxillary canines (*p* = 0.004); while mandibular canines and incisors were not found to be filled in the present study. The tooth-based comparison of dmft scores is shown in Table 4.

The arch-based comparison of tooth-type dmft scores is summarized in Table 5. The maxillary incisors were observed with a higher dmft score than mandibular incisors, with a statistically significant difference (*p* < 0.05). The mean dmft scores were higher for maxillary canines than mandibular canines; however, the comparison was not statistically significant (*p* > 0.05). The mean scores were less for maxillary molars compared to mandibular molars, with statistically significant differences (*p* < 0.05).

The result of the multivariate analysis is shown in Table 6. Four variables in the final model were analyzed, including sex, age group, tooth busing frequency, and previous dental visits. The multivariate regression analysis showed that children’s age and dental visits have a significant effect on mean dmft scores (*p* < 0.05), whereas sex and tooth brushing frequency was not found to have any impact on the mean dmft scores (*p* > 0.05). The multivariate regression analysis showed that children below 3 years showed significantly lower mean dmft scores compared to 3.1–6 years and above 6 years of age. Similarly, children who never visited had lower mean scores than children with previous dental visits (*p* < 0.05). However, study participants’ age and last dental visit were found to have a significant correlation with mean dmft scores (*p* < 0.05).

## 4. Discussion

Dental caries is one of the commonest and perhaps most prevalent diseases affecting the oral cavity [3,4,5,6,7,8,9,10]. It is a microbial disease of the calcified tissues of the teeth, characterized by demineralization of the inorganic component and destruction of the organic content of the tooth [2]. Many factors, including age, sex, ethnicity, dietary habits, and oral hygiene routines, affect the prevalence and incidence of dental caries in a community [8,9,10,11,12]. The present study attempted to establish a relationship between dental caries status and tooth brushing habits, and previous dental visits. A recent systematic review estimated that dental caries prevalence among 5–7 years old was 84% [18], and based on this review, the sample size was estimated. The study sample was selected from the children attending the pediatric specialty clinic at a teaching hospital and employed based on inclusion and exclusion criteria. Although the sample size was only modest, the surveyed children should be relatively representative of Saudi children with primary dentition. Various studies [19,20,21,22,23,24,25,26,27,28,29,30,31,32,33,34,35,36] reported on dmft in Arabian children; among these studies, few researchers focused only on primary dentition [19,27,28,29,30,31,32,33], and some concentrated on both primary and permanent dentitions [20,21,22,23,24,25,26,34,35,36]. The reported prevalence of carious in primary teeth ranges from 57.2% to 91.2% from previously reported studies in the literature [19,20,21,22,23,24,25,26,27,28,29,30,31,32,33,34,35,36]. However, according to the present study, the dental caries prevalence in primary teeth was 78.8% of 268 Arabian children with a mean age of 4.6 ± 1.1 years. Alamoudi et al. [28] reported a 73.5% carious prevalence among 517 children of 6–7 years old from Jeddah, and the sex of the subjects was not reported. Alamri et al. [30] reported a 76.4% prevalence among 672 male children belonging to the 6 years of age group. These two studies reported a similar prevalence of dental caries as the present study; however, the findings were not comparable because one study did not mention sex, the other research was conducted in only male children, and the present study involved both sexes in the study. Another two studies [29,30] from Jeddah reported a higher prevalence of dental caries compared to the present study in both sexes, 83.4% and 87.1%, respectively. Alshiha et al. [32] reported an 85.5% caries prevalence among 6183 female subjects of 6–7 years from the Riyadh region. Consequently, another study reported an almost similar prevalence (83%) in 578 male children from the Riyadh region of 6.92 years mean age [19]. Nevertheless, these studies’ findings are not comparable with the present study.

The mean dmft score was 5.82 ± 4.48, observed in the present study, while the overall mean scores for decayed, missing, and filled teeth were 3.903 ± 3.69, 1.18 ± 1.69, and 0.73 ± 1.35, respectively. Wyne et al. [37] reported a lower dmft of 2.92 ± 3.51 of 322 Arabian preschool children from the Alhasa region of Saudi Arabia. These findings are comparatively low when compared to the present study. Subsequently, Paul et al. [33] observed higher 7.1 dmft scores in 103 Arabian school children from the Alkarj region compared to the present study. The major research from Saudi Arabia on dental caries status was reported from Jeddah by Gandeh and Milaat [29], but the authors did not report the dmft scores among their study population. Another study [31] reported higher dmft mean scores in Saudi children (5.37 ± 3.60) compared to non-Saudi children (5.13 ± 3.55) residing in Saudi Arabia. However, in the present study, only school children with the primary dentition of Saudi nationality were involved.

Tooth brushing habits, in particular, have been shown to significantly contribute to a decrease in the prevalence of dental caries [37,38,39,40,41,42]. It has been found that there is a correlation between the act of brushing one’s teeth and the absence of dental caries [8,21,25]. Only 66.4% of participants in this study brushed their teeth at least once, and only 15.7% brushed more than once. Despite this, Farooqi et al. [21] found that almost 81 percent of the children brushed their teeth daily, with 61 percent reporting that they brushed their teeth twice a day. The present study revealed a correlation between tooth brushing habits and higher dmft scores that were found to be statistically significant. Children brushing their teeth regularly had better dmft scores than those children who have not brushed their teeth regularly. Similarly, Farooqi et al. [21] reported that good brushing habits were associated with a low incidence of dental caries. However, the results of their study were not statistically significant. According to the findings of the present study, Arabian children who had never brushed their teeth before had significantly higher mean dmft scores (7.5 ± 4.56) compared to those who rarely brushed (4.10 ± 4.38) and children who brushed their teeth once a day (6.06 ± 4.27), with statistically significant (*p* < 0.001) results. The mean dmft scores of the children who had brushed their teeth at least once were significantly lower than those who had either never brushed their teeth or only done so infrequently. Previous studies carried out in China [39], Hong Kong [40,41], and Ireland [42] substantiate the findings of the present study. Late brushing habit adoption and irregular brushing habits are also possible reasons for the higher prevalence of dental caries among Arabian school children [19], as shown in the present study. Another study also reported that children with poor brushing habits had higher caries rates and plaque deposits [33]. These findings are in agreement with the present study; however, the present study only compared tooth brushing habits and dmft scores. According to the American Association of Pediatric Dentistry (AAPD) [43], it is the responsibility of the dental profession to encourage brushing from the time of the eruption of primary teeth, eventually assisting children in reducing the caries burden. This should begin at the time of the eruption of the primary teeth. When fluoride toothpaste is used, brushing the teeth can help to deliver fluoride to the tooth surfaces, which can help to keep the teeth clean [44,45,46,47]. The results of the present study suggested that tooth brushing habit is one of the significant factors that can have an effect on the dmft scores of the child.

Thomson et al. [48] reported that the notion of having regular dental visits might be associated with better oral health. Children who visit the dentist on a regular basis may have better oral hygiene, fewer dental problems, and control over the number of sugary drinks they consume [49]. In the previous study, it was evident that children who visited had higher dmft scores (8.14 ± 3.12) compared to the children who had never been to the dentist in the past, who had lower dmft scores (4.71 ± 4.61). This could be because the children who needed treatment or complained went to the dentist. This concept was supported and postulated by a few researchers [48,49,50,51,52]. Visiting the dentist not only has a therapeutic component, but the educational intervention performed by health professionals within their practice also helps promote oral health in the population [51,52,53,54]. Going to the pediatric dentist can help you achieve this goal in a number of ways, including the therapeutic and educational benefits that it provides. Children who are at a higher risk of developing dental disease should be given priority for preventative dental care prior to the age of three [54]. After the eruption of the first tooth in the oral cavity, it is very important to take children to the pediatric dentist on a regular basis.

The prior studies [37,55] reported that mandibular first molars were the most frequently affected by carious lesions among Arabian children, followed by mandibular second molars and maxillary central incisors in the primary dentition. A Tanzanian study on 546 preschoolers living in urban areas reported similar findings among the primary teeth [56]. Paul et al. [33] reported that molars are commonly affected by tooth decay in Arabian children. In contrast, the present study found that tooth decay most commonly affects the mandibular second molars in the primary dentition. The scores for tooth base decay, missing, and filled cavities were also evaluated in the present study. The rate of caries was higher in the mandibular teeth than in the maxillary teeth; these results were in contrast with those found in a Hong Kong study carried out among 700 children from southern China [57]. The authors reported that the maxillary central incisor had a higher caries prevalence in their study. In contrast, the present study found that mandibular molars had a higher caries prevalence rate than other types of teeth in the oral cavity. On the basis of the type of tooth, the mandibular right second molar was found to have higher dmft scores, while the mandibular central incisors were found to have lower dmft scores.

There are numerous methods reported for preventing dental caries in children with primary dentition [58,59,60]. Parental education on preventive approaches to dental caries was also reported to have a significant role in reducing caries burden among their children [61,62]. Regular dental visits, tooth brushing with fluoridated toothpaste, and frequent professional fluoride applications may have a significant role in reducing the caries burden among children in primary dentition [63,64,65]. Children need the support of their families and caregivers as they learn to choose and maintain healthy lifestyle habits, including proper nutrition and dental care [66]. There is a need for early intervention programs targeting preschoolers’ verbal health behavior based on the risk variables found in the present study. Improving the quality and access to oral healthcare requires the involvement of policymakers.

### Limitations

Although the study is being conducted within a hospital, we have included all children who are in their primary dentition and are of Saudi nationality. Paul et al.’s [33] study included the participation of 103 children; some researchers used only male subjects, while others used only female subjects. In spite of this, the participants in the present study were of both sexes. Only sex, age, frequency of tooth brushing, and the number of previous dental visits were compared in this study. This also takes into consideration a possible restriction. The majority of the studies focused on aspects such as oral hygiene, parental education, socioeconomic background, body mass index (BMI), changes in salivary flow, twin studies, and other related topics. On the other hand, the present study is one of the few studies that focused exclusively on tooth brushing habits and previous dental visits. It is interesting to note that the present study found a negative association between tooth brushing habits and dmft scores. On the other hand, a positive association was found between dmft scores and previous dental visits. The comparison based on sex and age demonstrated a positive and statistically significant association with mean dmft scores.

## 5. Conclusions

Dental caries were found to be prevalent among Arabian children at a rate of 78.8%, with a mean dmft of 5.82 ± 4.48. Mandibular second molars were commonly affected by dental caries, while mandibular central incisors were less frequently affected. Dental caries was found to have a positive link with both age and frequency of visits to the dentist, but no correlation was observed between dental caries and sex or frequency of tooth brushing. The study findings highlight the need for education for both children and their parents in addition to the development of general and oral health interventions for children.

## Figures and Tables

**Table 1:** Comparison of dmft scores based on gender, age, tooth brushing, and previous dental visit.

Independent Variable	Group	n (%)	dmft Mean (SD)	*p*-Value
Sex	Male	178 (66.4)	4.97 (4.39)	<0.001 *
	Female	90 (33.6)	7.51 (4.18)	
Age	1–3	27 (10.1)	1.74 (2.33)	<0.001 *
	3.1–6	205 (76.5)	6.58 (4.28)	
	>6	36 (13.4)	4.58 (4.91)	
Tooth brushing frequency	Once	136 (50.7)	6.06 (4.27)	<0.001 *
	Twice	42 (15.7)	3.90 (4.07)	
	Never	60 (22.4)	7.50 (4.56)	
	Rarely	30 (11.2)	4.10 (4.38)	
Previous dental visit	Yes	87 (32.5)	8.14 (3.12)	<0.001 *
	No	181 (67.5)	4.71 (4.61)	

d = decayed; m = missing; f = filling; t = teeth; * *p* < 0.05 statistically significant.

**Table 2:** The details of dmft scores based on tooth type.

ToothNo/dmft	55	54	53	52	51	61	62	63	64	65	75	74	73	72	71	81	82	83	84	85
d (N)	125	76	34	34	35	55	30	29	71	101	110	74	17	8	10	7	7	26	88	128
m (N)	0	31	1	27	66	59	33	0	35	0	5	29	0	0	0	0	0	0	30	9
f (N)	14	9	2	1	3	3	0	1	10	25	33	29	5	0	0	0	0	5	22	40
dmft (N)	139	116	37	62	104	114	63	30	116	126	148	132	22	8	10	7	7	31	140	177

d = decayed; m = missing; f = filling; t = teeth; Tooth numbering based on FDI notation.

**Table 3:** Comparison of dmft based on gender in different age groups.

	<3 Years			3.1 to 6 Years			>6 Years		
dmft	Male	Female	*p*-Value	Male	Female	*p*-Value	Male	Female	*p*-Value
	Mean (SD)			Mean (SD)			Mean (SD)		
dmft	1.47 (2.56)	2.38 (1.59)	0.054	5.77 (4.32)	8.01 (3.85)	0.001 *	3.61 (4.28)	8.00 (5.68)	0.075
d	0.95 (2.04)	1.63 (1.68)	0.135	3.69 (3.24)	5.78 (3.92)	<0.001 *	2.11 (2.79)	5.63 (6.05)	0.095
m	0.42 (0.84)	0.63 (0.92)	0.465	1.34 (1.80)	1.24 (1.71)	0.886	0.93 (1.56)	1.38 (1.84)	0.448
f	0.11 (0.46)	0.13 (0.35)	0.559	0.74 (1.34)	0.99 (1.45)	0.101	0.57 (1.47)	1.00 (1.93)	0.695

* *p* < 0.05 statistically significant.

**Table 4:** The comparison of dmft scores based on tooth type.

Tooth Type	D	M	F	dmft
	Mean (SD)	Mean (SD)	Mean (SD)	Mean (SD)
Maxillary incisors (52 + 51 + 61 + 62)	38.5 (11.2)	46.3 (19.1)	1.8 (1.5)	85.8 (27.2)
Maxillary canines (53 + 63)	31.5 (3.5)	0.50 (0.71)	1.5 (0.71)	33.5 (4.91)
Maxillary molars (55 + 54 + 64 + 65)	93.3 (24.9)	16.5 (19.1)	14.5 (7.3)	124.3 (10.9)
Mandibular incisors (72 + 71 + 81 + 82)	8.0 (1.4)	0 (0)	0 (0)	8.0 (1.4)
Mandibular canines (73 + 83)	21.5 (6.4)	0 (0)	0 (0)	26.5 (6.4)
Mandibular molars (75 + 74 + 84 + 85)	100 (23.8)	18.3 (13.1)	31.0 (7.5)	149.3 (19.6)
*p*-Value	0.004 *	0.025 *	0.003 *	0.003 *

* *p* < 0.05 statistically significant; Tooth No in FDI system.

**Table 5:** Comparison of tooth-based dmft mean scores based on arch type.

dmft	Maxillary Arch	Mandibular Arch	*p*-Value
	Incisors 52 + 51 + 61 + 62	Iandibular incisors 72 + 71 + 81 + 82	
Mean (SD)	85.7 (27.2)	10.2 (5.8)	0.020 *
	Canines 53 + 63	Canines 73 + 83	
Mean (SD)	33.5 (4.9)	26.5 (6.4)	0.439
	Molars 55 + 54 + 64 + 65	Molars 75 + 74 + 84 + 85	
Mean (SD)	124.2 (10.9)	149.2 (19.6)	0.042 *

* *p* < 0.05 statistically significant; Tooth No in FDI system.

**Table 6:** The relationship between dmft scores and independent variables was studied.

Independent Variable	Group	Beta (95% CI)	SE (Beta)	*p* Value
Sex	Male *			
	Female	1.04 (0.88, 1.13)	0.04	0.277
Age	1–3 *			
	3.1–6	1.38 (1.19, 1.61)	0.08	<0.001 *
	>6	1.46 (1.21, 1.75)	0.09	<0.001 *
Tooth brushing frequency	Once	1.12 (0.99, 1.26)	0.06	0.064
	Twice *			
	Never	1.11 (0.98, 1.27)	0.06	0.087
	Rarely	1.15 (0.97, 1.36)	0.09	0.114
Previous dental visit	Yes *			
	No	0.03 (0.01, 0.09)	0.55	<0.001 *

* *p* < 0.05 statistically significant.

## Data Availability

Data will be available upon request to corresponding authors.
